# Quantitative approaches underpinning decision making

**DOI:** 10.1002/bimj.201900202

**Published:** 2019-07-28

**Authors:** Martin Posch, Frank Bretz, Tim Friede, Georg Heinze

1

Innovative statistical approaches addressing the emerging challenges in the analysis of routine as well as clinical trial data are essential to foster medical progress and support policy making. In this spirit, the CEN‐ISBS Vienna 2017 – Joint Conference on Biometrics & Biopharmaceutical Statistics (www.cenisbs2017.org) brought together statisticians from academia, industry, and regulators under the theme “*Quantifying Life. Advancing Research. Enabling Decisions*”. The meeting took place at the Medical University of Vienna and was organized as a joint meeting of the Central European Network (CEN, consisting of the Austro‐Swiss, German, and Polish Regions of the International Biometric Society) and the International Society for Biopharmaceutical Statistics (ISBS). The conference included a symposium on regulatory statistics organized in collaboration with the European Medicines Agency.

This special issue comprises 16 articles ranging from the analysis of quality and preclinical data, clinical trials, agricultural statistics, to advanced statistical methods for observational studies. Erhardt et al. investigate a Bayesian method to integrate information of in vivo and in vitro studies and Hoffelder assesses the use of the Mahalanobis distance to demonstrate equivalence of dissolution profiles (see also the corresponding letter to the editor of Collignon, Moellenhoff, & Dette (Biometrical Journal 2019, 61(3), 779–782) discussing regulatory implications of the proposal and Hoffelder's reply, in this issue). Gould proposes a Bayesian model averaging approach for early dose finding studies. The papers on Bayesian basket (Xu et al.) and adaptive clinical trials (Hsiao, Liu, & Mehta) address challenges in the design and analysis of such trials. Schmidtmann, Konstantinides & Binder assess the efficiency of the Wilcoxon–Mann–Whitney test for clinical trials where some patients die before the endpoint can be observed. The use of Bayesian models to aggregate information from several clinical trials with binary endpoints is investigated in the studies of Gravestock & Held and Chen & Lee. Bhattacharyya et al. summarize the findings of a panel discussion on current challenges for data monitoring committees held at the CEN‐ISBS 2017 and comprise academic, industry, and regulatory perspectives. Al‐Sarraj, Brömssen, & Forkman study the construction of prediction intervals in random effects models, a problem motivated by agricultural experiments. Challenges arising in observational studies are addressed in articles on estimation in the presence of multiple lower limits of quantification (Berger, Hilgers, & Heussen), the selection of covariates in the context of causal inference (Witte & Didelez), and in contributions on complex time to event (Rousson et al.) or multistate models (James et al.). Last but not least, Boulesteix et al. take up the topic of reproducible research in the application of random forest prediction models, evaluate the current practice, and formulate recommendations.

Apart from the scientific program of the CEN‐ISBS 2017, we enjoyed the opportunities for informal exchange and discussions, last but not least at the welcome reception at the Great Ball Room of Vienna's city hall. We thank all sponsors and supporters of the conference and especially all reviewers of this special issue for their time and expertise.

Finally, we look forward to the next ISBS conference in Kyoto in 2019 and the CEN/GMDS meeting in Berlin in 2020. See you there!

